# Differential effects of low and high temperature stress on pollen germination and tube length of mango (*Mangifera indica* L.) genotypes

**DOI:** 10.1038/s41598-023-27917-5

**Published:** 2023-01-12

**Authors:** Xinyu Liu, Yilin Xiao, Jing Zi, Jing Yan, Chunhong Li, Chengxun Du, Jiaxin Wan, Hongxia Wu, Bin Zheng, Songbiao Wang, Qingzhi Liang

**Affiliations:** 1 Key Laboratory of Tropical Fruit Biology, Ministry of Agriculture and Rural Affairs of China/Key Laboratory of Hainan Province for Postharvest Physiology and Technology of Tropical Horticultural Products, South Subtropical Crops Research Institute, Chinese Academy of Tropical Agricultural Sciences, Zhanjiang, 524000 China; 2 Renhe Meteorological Bureau of Panzhihua, Panzhihua, 617000 China; 3 College of Agriculture, Guangxi University, Nanning, 530000 China

**Keywords:** Ecology, Physiology, Plant sciences

## Abstract

Mango flowering is highly sensitive to temperature changes. In this research, the maximum values of pollen germination rate (PGR), pollen tube length (PTL) and their cardinal temperatures (Tmin, Topt and Tmax) were estimated by using quadratic equation and modified bilinear model under the conditions of 14–36 °C. The pollen germination rate in four mango varieties ranged from 29.1% (‘Apple mango’) to 35.5% (‘Renong No. 1’); the length of pollen tube ranged from 51.2 μm (‘Deshehari’) to 56.6 μm (‘Jinhuang’). The cardinal temperatures ranges (Tmin, Topt and Tmax) of pollen germination were 20.3–22.8 °C, 26.7–30.6 °C and 30.4–34.3 °C, respectively; similarly, cardinal temperatures (Tmin, Topt and Tmax) of pollen tube growth were 20.3–21.2 °C, 27.9–32.1 °C and 30.2–34.4 °C respectively. Of those, ‘Renong No. 1’ could maintain relatively high pollen germination rate even at 30 °C, however, ‘Deshehari’ had the narrowest adaptive temperature range. These results were further confirmed by changes of superoxide dismutase, catalase activity and malondialdehyde content. These results showed that mango flowering was highly sensitive to temperature changes and there were significant differences in pollen germination rate and pollen tube length among different varieties. Current research results were of great significance for the introduction of new mango varieties in different ecological regions, the cultivation and management of mango at the flowering stage and the breeding of new mango varieties.

## Introduction

Mango, known as the ‘king of tropical fruits’, is one of the most popular fruits in the world^1^. Mango is widely cultivated in tropical and warmer subtropical areas in the world. India, China, and Thailand are the top three producers. The global production of mango was 46.5 million tons in 2016, which ranks as the fifth most produced fruit crop worldwide (http://www.fao.org/faostat/). Mango fruits were mainly consumed fresh; only a small portion was processed like nectar, juice, jam, and powder and so on^1^. The fruits demonstrate attractive visual appearance and offer a favorable sensory experience to consumers, making them growingly popular among world consumers^2^.

Temperature has an important impact on plant growth and development, especially for flowering period^3,4^. Compared with vegetative processes, sexual reproduction of plants was more sensitive to high and low temperatures, and therefore, plant reproductive organs were more susceptible to short-term high and low temperature changes before and during flowering^5,6^. Many studies showed that temperature stress often led to pollen abortion and asynchronous development of pollen and stigma^7,8^, particularly, high temperature had significant effect on pollen germination, pollen tube growth, fertilization, flower abscission and fruit setting^9–11^; while low temperature inhibited pollen germination to decrease fruit setting rate^12^. In addition, the occurrence of extreme high temperature was frequent due to global warming in recent years^13^, which seriously affected pollen germination during flower period and fruit setting^14,15^, Global temperature may rise by 1.4–5.8 ℃ by 2100^16^, which will directly cause serious economic losses, therefore, more and more attention should be paid on the effect of pollen germination under temperature stress. Furthermore, it is very significant to identify and distinguish the response and tolerance of pollen to extreme weather for the sustainability of agriculture.

Pollen can be regarded as a favorable marker to characterize the tolerance of plant species or genotype due to specific responses^17^, as well as an independent functional unit once released from anther. Previous study suggested that both high temperature and low temperature stress mostly adversely affect the performance of pollen, such as reducing pollen germination rate and pollen tube length of different almond genotypes^18,19^. High temperature stress led to a decrease in pollen germination and pollen tube growth at different rates in different genotypes of peanut and coconut^20^. The response to low temperature induced germination and elongation of different genotypes of almond and sweet cherry was also different^10^. It can be concluded that the tolerance of different species or genotypes pollen can be evaluated by the differences of pollen germination rate and pollen tube growth under temperature stress^21^; tolerance of pollen under temperature stress was usually assessed by Cumulative Stress Response Index (CSRI)^22^. Therefore, the effect of pollen germination and pollen tube length under temperature stress is usually used to represent the relative sensitivity of different genotypes to stress conditions.

Meanwhile, the pollen tube was characterized by a polarized apical growth cell, which was controlled by the signal network composed of Ca^2+^, nitric oxide (NO) and reactive oxygen species (ROS)^23–25^. Among them, ROS regulated many cell growth processes such as pollen germination^25^, pollen tube elongation^26^, guidance and positioning of ovules^27^, and pollen tube rupture during fertilization^28^. ROS regulated stress responses and accumulate excessively under abiotic stress conditions^29^. Due to the excessive accumulation of ROS, antioxidants such as superoxide dismutase (SOD)^30^ and Peroxidase (POD)^31^ were balanced by scavenging mechanisms, thereby causing oxidative stress in organisms. In addition, excessive ROS accumulation led to lipid peroxidation of cell and organelle membranes and one product of lipid peroxidation was malondialdehyde (MDA)^32^. Therefore, the changes of SOD, POD activity and MDA content can be used as indicators to judge ROS tolerance under stress conditions and measure ROS damage to a certain extent.

In recent years, the area under mango plantation has expanded unceasingly worldwide, and mango production has increased year by year. Currently, more than 100 countries and regions worldwide produce mango, and China is one of the major mango-producing countries. The mango industry has become the mainstay of the local agricultural industry in most of the mango production provinces in China^33^. However, the global climate change such as frequent extreme climate in recent years, especially the late spring cold and dry hot wind in spring in China. It is necessary to study the effects of mango pollen under different temperatures stress. Four mango varieties ‘Renong No. 1’, ‘Jinhuang’, ‘Dashehari’ and ‘Apple mango’, those were the main promoted varieties in different provinces in South China (‘Dashehari’ was mainly cultivated in Zhanjiang City, Guangdong Province; ‘Jinhuang’ was the main variety promoted in Guangxi and Yunnan provinces; ‘Apple mango’ was mainly grown in the eastern part of Guangdong Province and Fujian Province; ‘Renong No. 1’ was bred by Chinese Academy of Tropical Agricultural Sciences, which can be grown in both tropical and subtropical regions of South China) were used to research pollen germination rate (PGR), pollen tube length (PTL) and their cardinal temperatures (T_min_, T_opt_ and T_max_) under temperature stress. This research combined the different temperature stress with cumulative stress response index (CSRI) by using the method of germination in vitro to confirm the optimum temperature during the mango flowering period and the effect of pollen vitality under high and low temperature stress, so as to quantify the response of pollen germination and pollen tube growth to temperature stress in different mango varieties, as well as the cardinal temperature of mango varieties. These results lay the foundation for the reproductive biology research and breeding of mango, furthermore, which can be useful for selecting the best varieties in different mango growing regions.

## Results

### Response of pollen germination and pollen tube growth under temperature stress

#### Response of pollen germination under temperature stress

**Figure 1:**
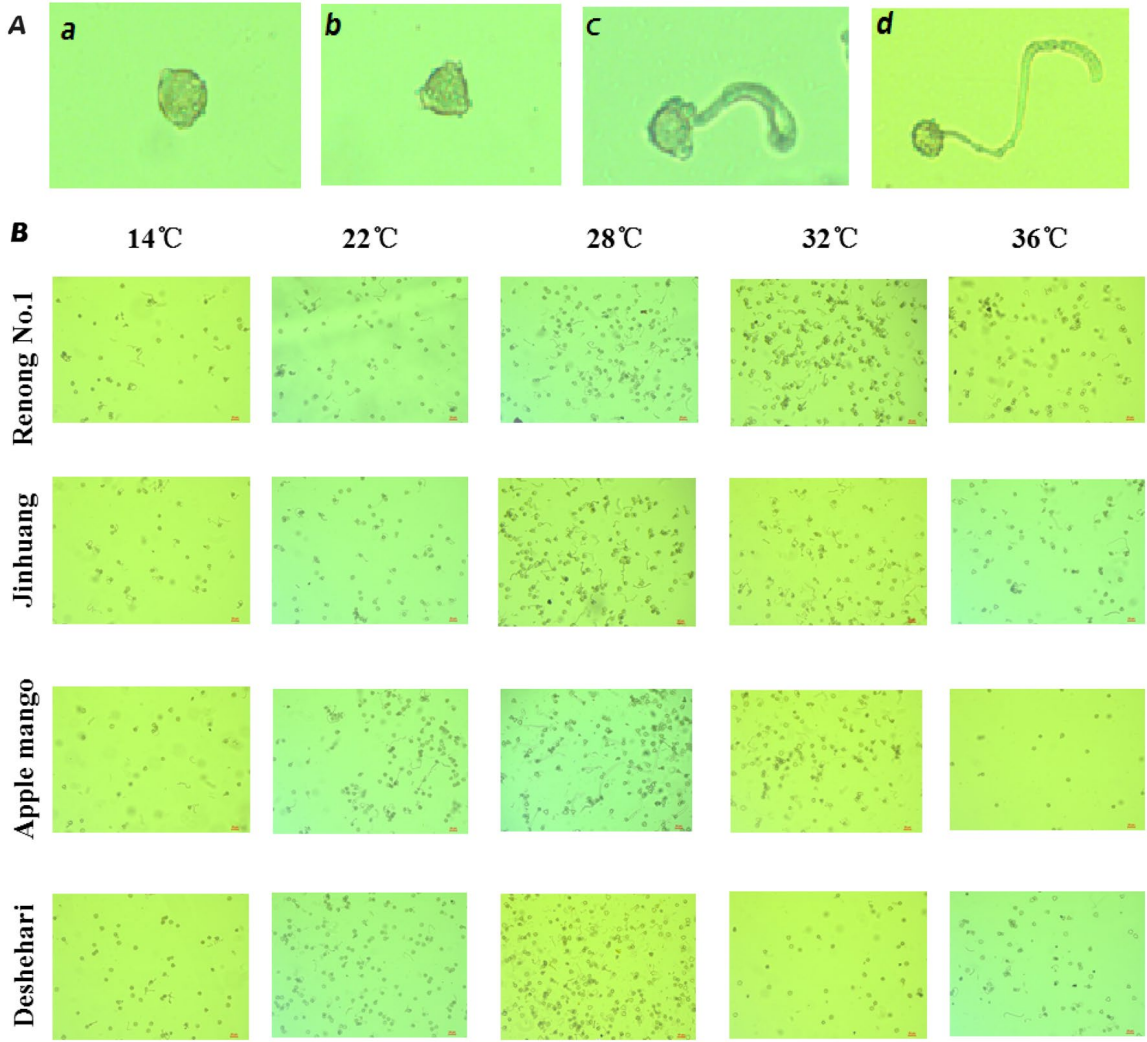
Pollen germination stage at different temperatures. (**A**) (**a**) No germination, (**b**) ready for germination, (**c**) in germination, (**d**) germinated; (**B**) field of view of pollen germination of different varieties under different temperatures stress.

**Figure 2:**
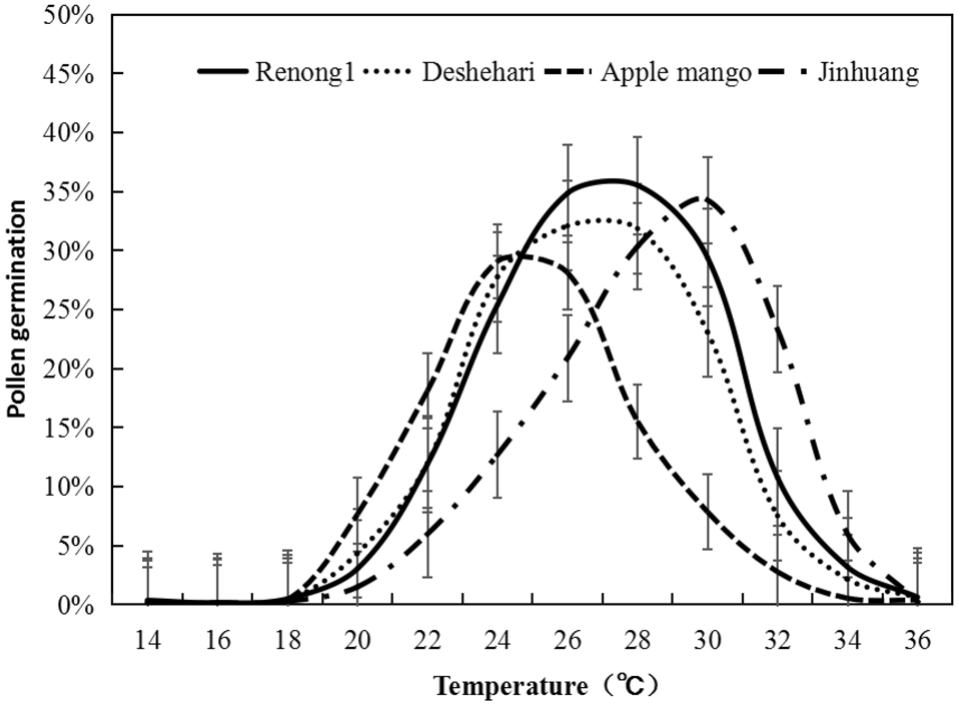
Pollen germination of four mango genotypes in response to different temperature stress.

**Figure 3:**
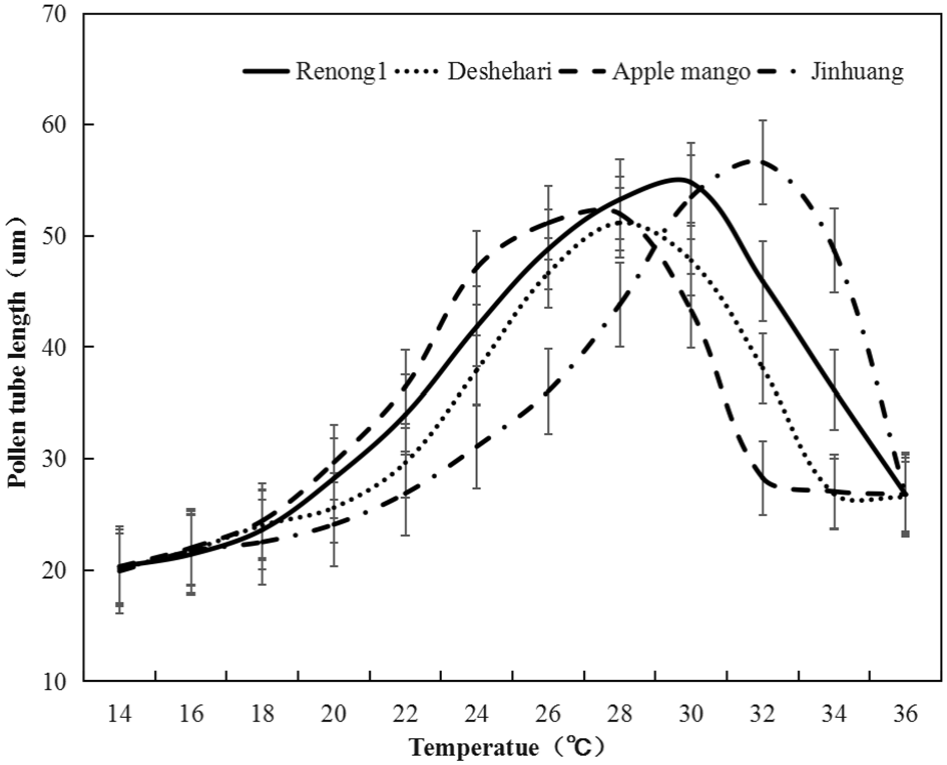
Pollen tube length of four mango genotypes responsed under different temperature stress.

The preliminary experiments results on pollen germination and pollen tube growth of different varieties at different temperatures (Fig. 1) showed that: Overall, after all mango varieties were cultured at 24 ℃ ~ 30 ℃ for 3 h, the pollen germination rate and pollen tube length reached the maximum. Pollen was difficult to germinate below 20 ℃, and then the pollen germination growth rate of most varieties of mango gradually increased with the increase of temperature, reaching the maximum at 28 ℃, with an average germinate rate of 28.3%, and then decreased, the pollen germination rate was less than 10% when the temperature was higher than 32 ℃ (Fig. 2; Table 1). The growth of pollen tube also indicated similar trend, from the average value of 50.1 μm at 28 ℃ dropped to 34.7 μm at 34 ℃ (Fig. 3; Table 2).

**Table 1:** Maximum pollen germination percentage and cardinal temperatures for pollen germination of four mango genotypes in response to different temperature stress.

Cultivar	Max pollen germination (%)	Cardinal temperature		
		T_min_	T_opt_	T_max_
Renong No. 1	35.5	22.1	30.6	33.7
Deshehari	32.1	21.9	28.3	32.6
Apple mango	29.1	20.3	26.7	30.4
Jinhuang	34.3	22.8	30.4	34.3
Mean	32.8	21.8	29.0	32.8
s.e.d	3.07*	0.29**	1.61**	1.71**

*P < 0.05, **P < 0.01.

**Table 2:** Maximum pollen tube length and cardinal temperatures for pollen tube length of four mango genotypes in response under different temperature stress.

Cultivar	Max pollen tube length (μm)	Cardinal temperature		
		T_min_	T_opt_	T_max_
Renong No. 1	54.8	20.5	30.4	33.8
Deshehari	51.2	20.7	28.3	30.3
Apple mango	52.0	20.3	27.9	30.2
Jinhuang	56.6	21.2	32.1	34.4
Mean	53.7	20.7	29.7	32.2
s.e.d	2.17**	0.39**	1.69**	1.65**

*P < 0.05, **P < 0.01.

The quadratic equation (Eq. 1) described the response of pollen germination under temperature stress (R^2^ > 0.84). As shown in Fig. 2, the pollen germination rate of mango obviously involved with temperature and variety. The maximum pollen germination rates of the four species were as followed: ‘Renong No. 1’ 35.5%, ‘Dashehari’ 32.1%, ‘Apple mango’ 29.1%, ‘Jinhuang’ 34.3%, respectively, with the average value of 32.8%. Meanwhile, the basic temperature of different varieties was various. T_min_ ranged from 20.3 ℃ (‘Apple mango’) to 22.8 ℃ (‘Jinhuang’); T_opt_ ranged from 26.7 ℃ (‘Apple mango’) to 30.6 ℃ (‘Renong No. 1’); T_max_ ranged from 30.4 ℃ (‘Apple mango’) to 34.3 ℃ (‘Jinhuang’); the temperature ranged (T_max_ − T_min_) from 10.1 ℃ (‘Apple mango’) to 11.6 ℃ (‘Renong No. 1’). According to Table 1, it was found that ‘Renong No. 1’ pollen had the highest the germination rate and optimum temperature, as well as the widest temperature adaptability, even may maintain a relatively high pollen germination rate at 30 ℃; While the smallest pollen germination rate of ‘Apple mango’ referred to poor adaptability to the flower period temperature.

### Response of pollen tube growth under temperature stress

There were also significant differences among the response of pollen tube length under different temperature stress for four mango varieties (Fig. 3), similar to the response of pollen germination rate to different temperature stress, as the modified bilinear model described (R^2^ > 0.93). At the optimum temperature, the length of pollen tube was as follows: ‘Renong No. 1’ 54.8 μm, ‘Dashehari’ 51.2 μm, ‘Apple mango’ 52.0 μm, ‘Jinhuang’ 56.6 μm, with the average value of 53.7 μm. T_min_ ranged from 20.3 ℃ (‘Apple mango’) to 21.2 ℃ (‘Jinhuang’), T_opt_ ranged from 27.9 ℃ (‘Apple mango’) to 32.1 ℃ (‘Jinhuang’), T_max_ ranged from 30.2 ℃ (‘Apple mango’) to 34.4 ℃ (‘Jinhuang’). The temperature ranged (T_max_ − T_min_) from 9.6 ℃ (‘Dashehari’) to 13.3 ℃ (‘Renong No. 1’). According to Table 1, it was found that the ‘Dashehari’ pollen tube had the narrowest range of temperature, and the shortest growth length at the optimum temperature, which largely affected the fruit set and yield of ‘Dashehari’.

**Figure 4:**
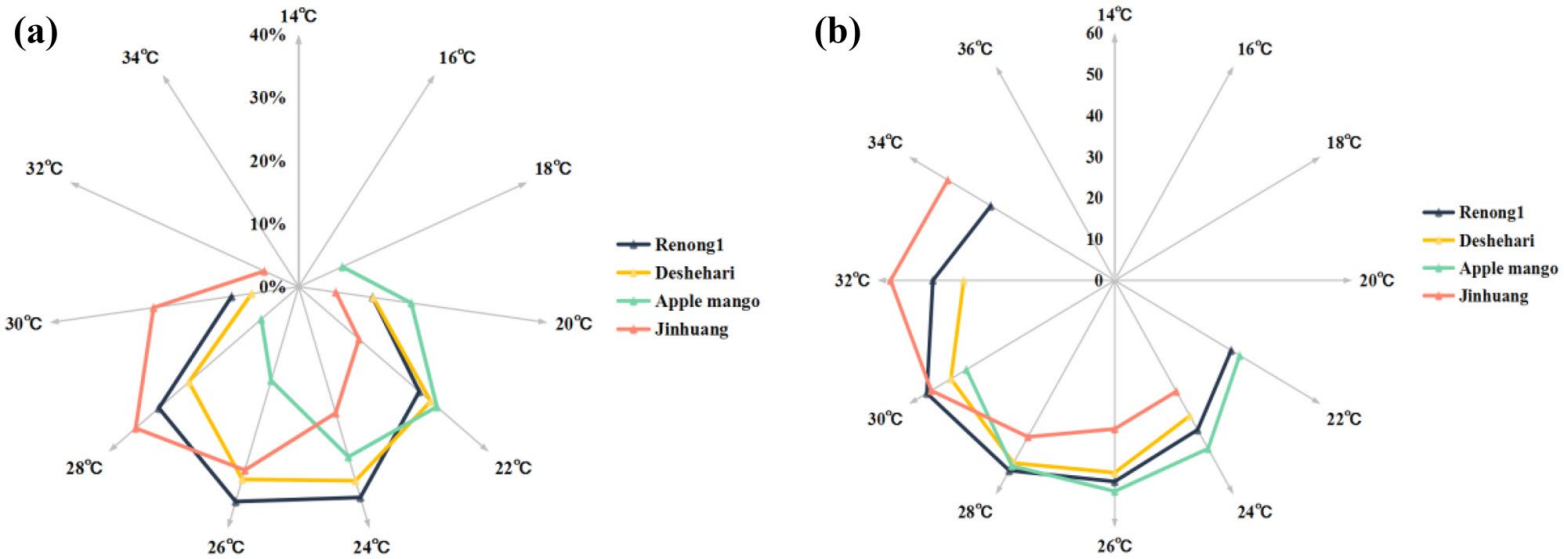
Differences of pollen germination rate (**a**) and pollen tube length (**b**) of four mango genotypes.

A pollen germination rate of more than 5% was usually considered efficient germination. In terms of the pollen germination rates of four mango varieties, the effective germination temperature of ‘Apple mango’ was about 20 ℃–30 ℃, and ‘Jinhuang’'s about 24 ℃–34 ℃. Among the adaption temperature of the four varieties for effective germination, ‘Apple mango’ was lowest, and ‘Jinhuang’ was highest; ‘Dashehari’ and ‘Renong No. 1’ were about 22 ℃–32 ℃, however, within the same temperature range, the pollen germination rate of ‘Renong No. 1’ was higher than that of ‘Dashehari’, and the difference became more significant as the temperature increased. (Fig. 4a).

Pollen tube length over 30 μm was usually regarded as the effective growth, based on the pollen tube length of four mango genotypes, the effective tube growth temperature of ‘Apple mango’ was about 22 ℃–30 ℃, and 24 ℃–32 ℃ for ‘Dashehari’, both the temperature range was 8 ℃. Meanwhile, ‘Jinhuang’ was about 24 ℃–34 ℃, and 22 ℃–34 ℃ for ‘Renong No. 1’, both the highest temperature reached 34 ℃. However, ‘Renong No. 1’ had largest the temperature adaptability range, which was 12 ℃, followed by ‘Jinhuang’ with 10 ℃. These results suggested ‘Renong No. 1’ had a wide range of temperature adaptability (Fig. 4b).

### Pollen germination and culture time in vitro

**Figure 5:**
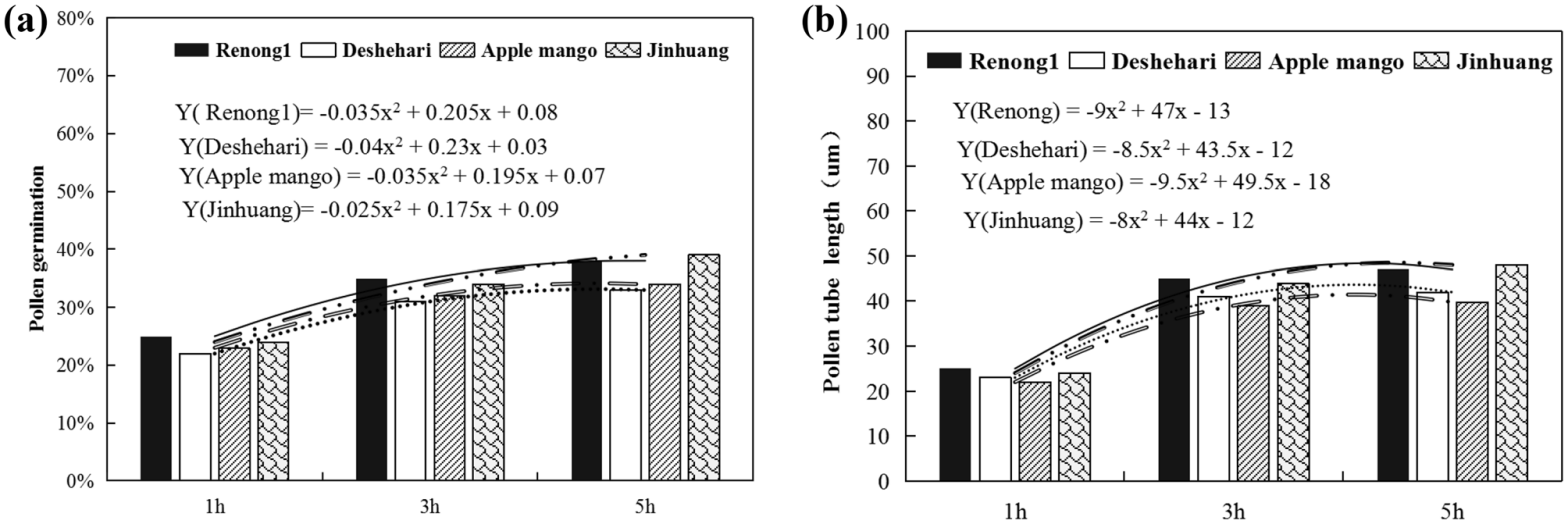
Variation of pollen germination rate (**a**) and pollen tube length (**b**) of four mango varieties with different culture time.

Pollen culture in vitro for 1 h, 3 h and 5 h at room temperature showed that pollen germination was obvious with the increase of culture time on the culture medium. The pollen germination rate of the four varieties increased sharply within 3 h before culture, and there was no significant difference among the varieties. However, after 5 h of culture, the growth rate of pollen germination of all varieties decreased significantly, and the change of pollen tube length with culture time also showed a similar trend (Tables 3 and 4). On the whole, ‘Jinhuang’ and ‘Renong No. 1’ were more tolerant to long-term temperature than ‘Dashehari’ and ‘Apple mango’ (Fig. 5).

**Table 3:** Growth rate of pollen germination of four mango varieties at different culture time.

Varieties	Time	
	1–3 h	3–5 h
Renong No. 1	0.40	0.09
Deshehari	0.41	0.06
Apple mango	0.39	0.06
Jinhuang	0.42	0.15

**Table 4:** Growth rate of pollen tube length of four mango varieties at different culture time.

Varieties	Time	
	1–3 h	3–5 h
Renong No. 1	0.80	0.04
Deshehari	0.78	0.02
Apple mango	0.77	0.02
Jinhuang	0.82	0.09

### Cumulative stress response index

**Figure 6:**
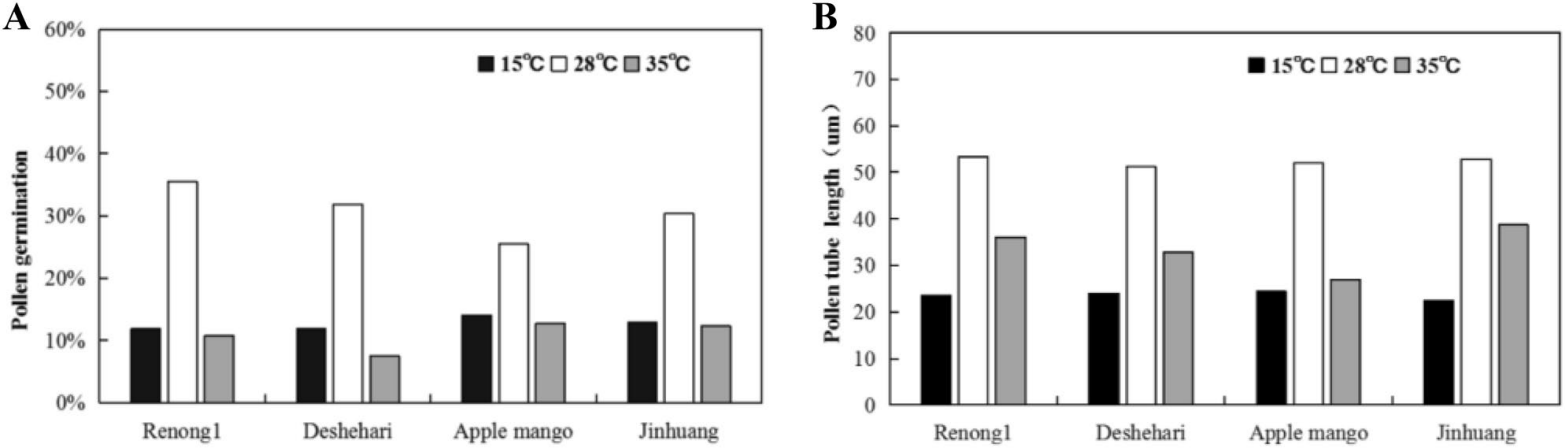
Germination rates (**A**) and pollen tube length (**B**) of pollen grains of four mango varieties at three different temperature conditions (15 °C, 28 °C and 35 °C).

In terms of the determination of pollen germination rate and pollen tube length, 28 ℃ temperature stress was used as control; the effects of germination rate under low temperature (15 ℃) and high temperature (35 ℃) were confirmed. The results showed that the germination rate of all mango varieties were higher at 28 ℃. Of them, the germination rate of ‘Renong No. 1’ was the highest, about 35.5%. The germination rates of ‘Renong No. 1’, ‘Dashehari’, ‘Apple mango’ and ‘Jinhuang’ decreased by 66.5%, 62.4%, 44.8% and 57.2% compared to those of under 28 ℃ in the low temperature stress, respectively. Besides, under high temperature stress, the germination rates of ‘Renong No. 1’, ‘Dashehari’, ‘Apple mango’ and ‘Jinhuang’ decreased by 69.7%, 76.4%, 50.0% and 57.2% compared to those of under 28 ℃, respectively. It can be seen that there was no significant difference between varieties under low temperature stress and high temperature stress, however, in terms of temperature change rate, high temperature stress had a greater impact on pollen germination rate (Fig. 6a).

Similar to pollen germination rate, the pollen tube length of all varieties were higher at 28 ℃ temperature stress, including the highest ‘Renong No. 1’, about 53.3 μm, However, under the stress of low temperature and high temperature, the growth of pollen tube degenerated. The pollen length of ‘Renong No. 1’, ‘Dashehari’, ‘Apple mango’ and ‘Jinhuang’ shorted by 55.7%, 53.1%, 53.0% and 57.4% compared to those of under 28 ℃ in the low temperature stress, respectively. Besides, under high temperature stress, the pollen tube length rates of 'Renong No. 1', ‘Dashehari’, ‘Apple mango’ and ‘Jinhuang’ were 32.1%, 35.9% and 47.9% and 26.8% compared to those of under 28 ℃, respectively. These results suggested that the inhibition effect of pollen tube growth under low temperature stress was significantly greater than that under high temperature stress (Fig. 6b).

In order to detect the pollen sensitivity and tolerance under low temperature and high temperature stress, the CSRI value was calculated (Table 5). According to CSRI data, ‘Renong No. 1’ was intermediate and ‘Dashehari’, ‘Jinhuang’ and ‘Apple mango’ were sensitive to low temperature. ‘Renong No. 1’ and ‘Jinhuang’ were intermediate genotype and ‘Jinhuang’, ‘Dashehari’ were sensitive to high temperature.

**Table 5:** CSRI values of four mango genotypes at different temperature stress.

Temperature	Renong No. 1	Deshehari	Apple mango	Jinhuang
15 ℃	−44.7	−104.9	−117.2.7	−98.3
Sensitive < −97.1 < intermediate < −78.7 < tolerant				
35 ℃	−59.6	−73.2	−82.7	−67.3
Sensitive < −71.3 < intermediate < −53.7 < tolerant				

### Response of SOD, POD enzyme activities and MDA content to temperature stress

**Figure 7:**
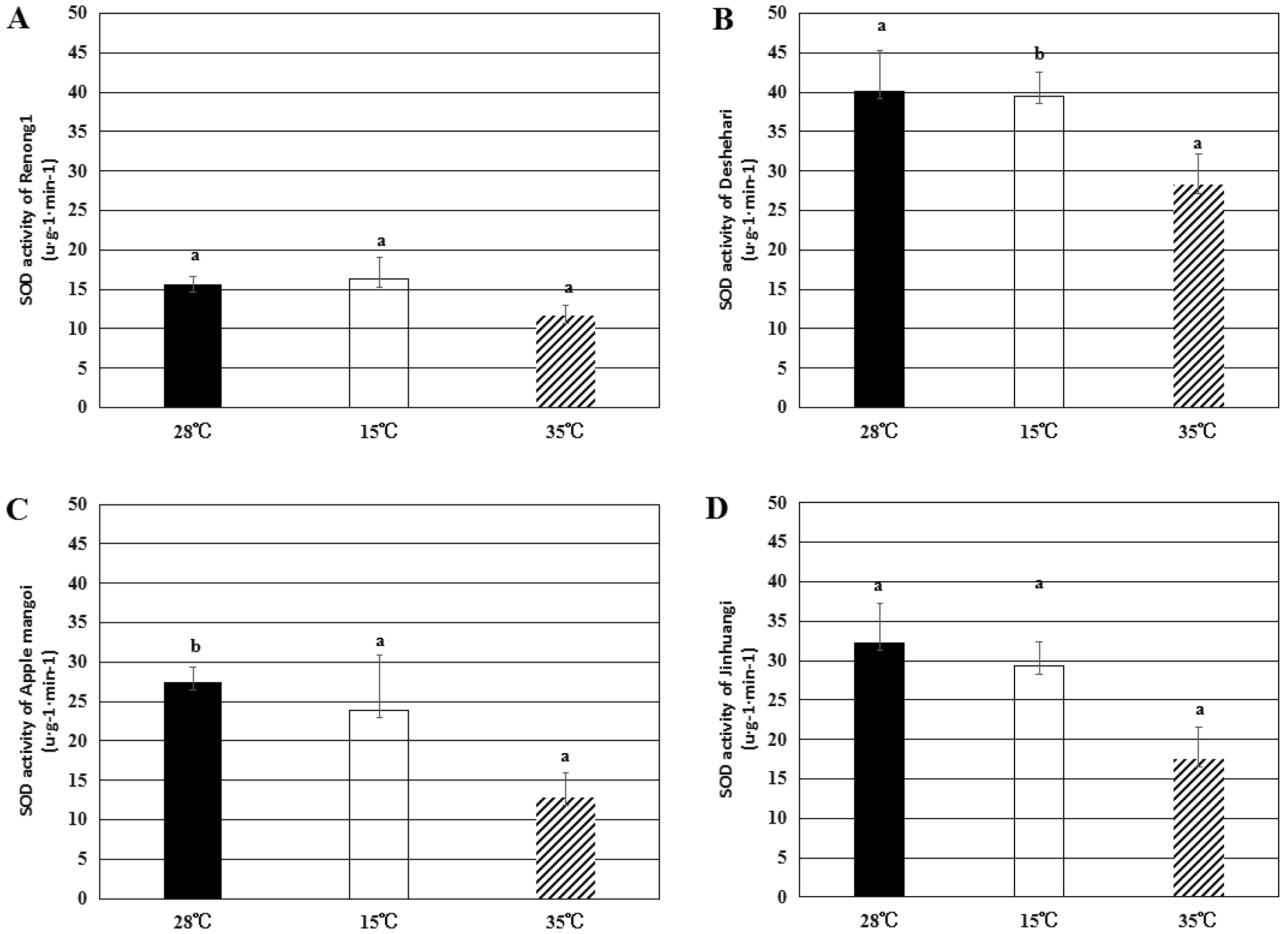
The SOD in pollen tubes of ‘Renong No. 1’ (**A**), ‘Deshehari’ (**B**), ‘Apple mango’ (**C**) and ‘Jinhuang’ (**D**) under three different temperature stress of 28 °C, 15 °C and 35 °C. Different letters represent statistically significant differences (P < 0.05), and error bars represent standard deviations.

**Figure 8:**
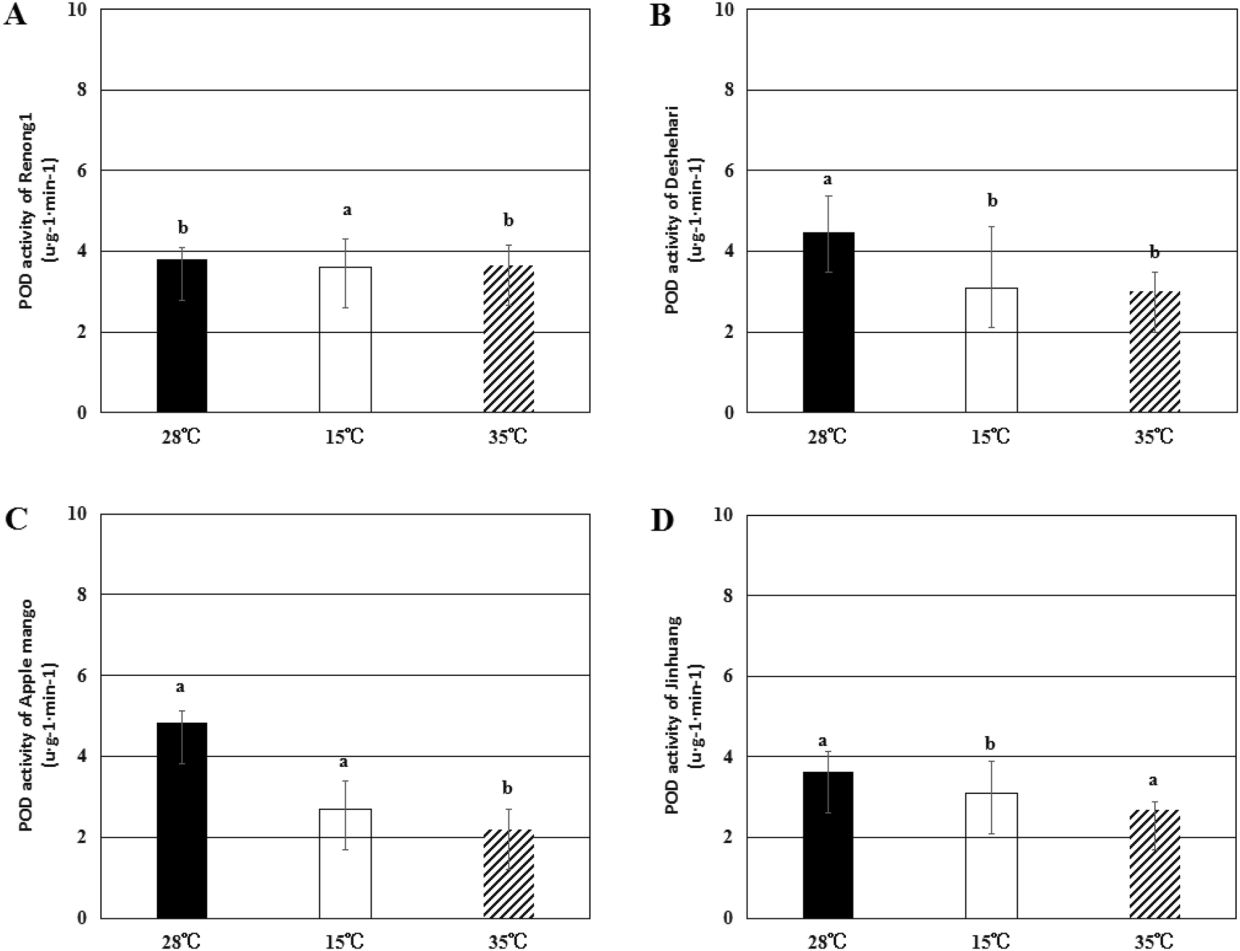
The content of POD in pollen tubes of ‘Renong No. 1’ (**A**), ‘Deshehari’ (**B**), ‘Apple mango’ (**C**) and ‘Jinhuang’ (**D**) under three different temperatures of 28 °C, 15 °C and 35 °C. Different letters represent statistically significant differences (P < 0.05), and error bars represent standard deviations.

**Figure 9:**
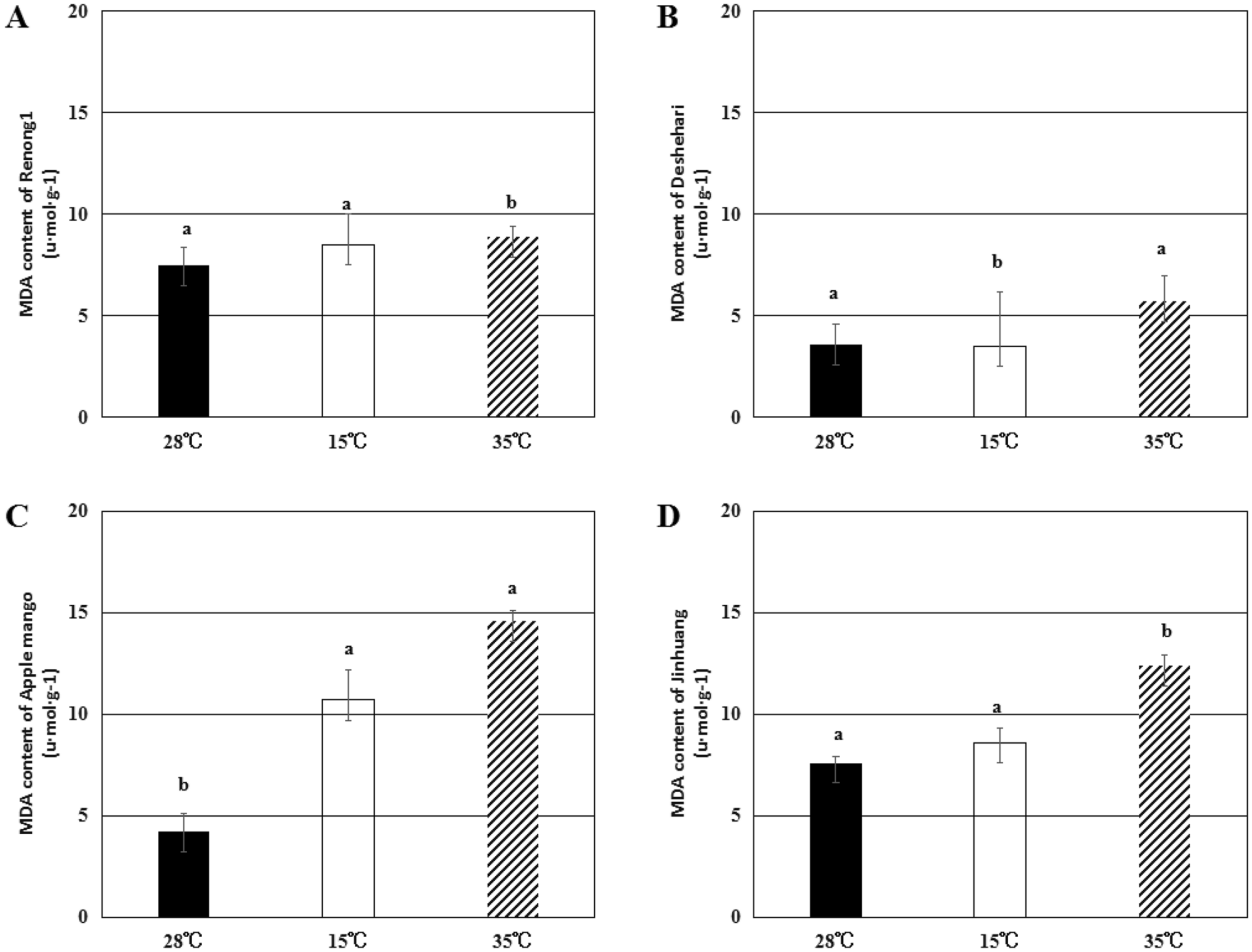
The content of MDA in pollen tubes of ‘Renong No. 1’ (**A**), ‘Deshehari’ (**B**), ‘Apple mango’ (**C**) and ‘Jinhuang’ (**D**) under three different temperatures of 28 °C, 15 °C and 35 °C. Different letters represent statistically significant differences (P < 0.05), and error bars represent standard deviations.

By measuring SOD and POD enzyme activities in pollen tubes, the response of pollen tubes to low temperature and high temperature stress was studied. The results showed that in the process of pollen germination, low temperature and high temperature treatments caused oxidative stress in pollen tubes, and high temperature had a more significant effect on pollen tubes than low temperature (Figs. 7, 8, 9).

The activity of protective enzymes was related to the ability of plants to adapt to adversity. Among them, SOD was a key enzyme in plant metabolism, which can maintain the normal metabolism of plants when free radicals were toxic to plants. After low-temperature treatment, the SOD activities of ‘Deshehari’, ‘Apple mango’ and ‘Jinhuang’ decreased by about 1.7%, 12.8% and 9.2%, respectively, while the SOD activity of ‘Renong No. 1’ increased by about 4.4%. Under high temperature conditions, the SOD activity of ‘Renong No. 1’ decreased by 25.1%, ‘Deshehari’ decreased by 29.9%, ‘Apple mango’ decreased by 52.9%, and ‘Jinhuang’ decreased by 45.8% (Fig. 7).

POD widely existed in plant cells and was a free radical scavenger, which can effectively reduce the damage to plants caused by adversity. Under low temperature conditions, the POD enzyme activity of ‘Renong No. 1’ decreased by about 5.1%, ‘Deshehari’ decreased by 30.6%, ‘Apple mango’ decreased by 44.1%, and ‘Jinhuang’ decreased by 14.4%. At high temperature stress, the POD activity of ‘Renong No. 1’ decreased by 3.7%, ‘Deshehari’ decreased by 32.9%, ‘Apple mango’ decreased by 54.4%, and ‘Jinhuang’ decreased by 25.4% (Fig. 8).

Under temperature stress conditions, damage to lipid membranes can be reflected by the level of MDA production. Low temperature stress increased the MDA content of ‘Renong No. 1’, ‘Apple mango’, and ‘Jinhuang’ by 13.3%, 154.8%, and 13.2%, respectively, and decreased by 2.8% in ‘Deshehari’. Under high temperature stress, the MDA content of ‘Renong No. 1’, ‘Deshehari’, ‘Apple mango’ and ‘Jinhuang’ increased by about 18.7%, 58.3%, 247.6% and 63.2%, respectively (Fig. 9).

## Discussion

With the change of global climate, scientists predicted that the global average temperature will rise by 1.4–5.8 ℃ by 2100^34^, as well as more acute temperature change, resulting in tremendous threat to growth of plant. This study aimed to explore the best mango genotype in different ecological zones by taking the determined pollen parameters as one of the parameters of breeding program due to the similar response trends of pollen germination rate in vitro and pollen tube length under temperature stress. However, because the basic temperature range among mango varieties was relatively narrow, it is necessary to further explore the germplasm resources of mango to determine the tolerance genotype of extreme weather. No matter the extreme climate at present than the trend of global warming in the future, it is necessary to develop new mango varieties with pollen to withstand extreme weather.

Temperature is a vital environmental factor that affects the plant reproductive process such as pollen germination, pollen tube growth and fruit setting and so on. This research results indicated that pollen germination rate and pollen tube length in vitro decreased seriously under high and low temperature stress, meanwhile the optimum temperature of four varieties were determined. Bilinear or modified bilinear regression models had been widely used to study the response of in vitro pollen germination to temperature and to screen heat-tolerant crops^35^. The observed pollen germination rates for cotton (44%)^18^, peanut (56%)^19^ and coconut (40%)^36^ under artificial medium were similar to the results of current study. Therefore, the response of pollen germination in vitro to temperature stress can be used as an accurate method to screen the best mango genotype in different ecological zones.

Among different mango varieties, the optimum temperature of pollen germination was significantly related with its maximum temperature. The highest optimum temperature of ‘Renong No. 1’ (30.4 ℃) also had the highest pollen germination rate (35.5%). Similar pollen condition occurred in maize^37^ and Cucumis melo^38^. Meanwhile, among the four mango varieties, ‘Renong No. 1’ had the widest temperature range (T_max_ − T_min_ = 11.6 ℃), followed by ‘Jinhuang’ (11.5 ℃). In the commercial varieties promoted in Southern China, ‘Renong No. 1’ had the largest temperature range and wider temperature adaptability, while ‘Apple mango’ had the smallest temperature range (10.1 ℃) and weaker temperature adaptability during flowering period. It was consistent with the main planting areas of the four mango varieties in China^39^. ‘Renong No. 1’ had the widest temperature range and the highest temperature, which can be cultivated in all (sub)tropical areas in southern China^40^; The optimum temperature of ‘Jinhuang’ was high, which was mainly planted in south subtropical region of Guizhou and Yunnan^41^; The temperature adaptability of ‘Apple mango’ was weak, so it cannot be widely used as the main variety in southern China; The optimum temperature of ‘Deshehari’ was high, but it was sensitive to temperature change. It was mainly planted in Zhanjiang city, Guangdong province and the yield was unstable due to interannual climate change.

The basic temperature of mango pollen germination was 21.8 ℃ (T_min_), 29.0 ℃ (T_opt_) and 32.8 ℃ (T_max_), respectively, it was similar to the cotton temperature of 14.0 ℃ (T_min_), 31.0 ℃ (T_opt_) and 43 ℃ (T_max_)^19^ as well as serpent cucumber 10 ℃ (T_min_), 30 ℃ (T_opt_) and 48 ℃ (T_max_)^38^. Meanwhile, among the four promoted mango varieties in different provinces in southern China, ‘Dashehari’ had the narrowest pollen tube growth temperature range and the shortest growth length at the optimum temperature, which largely affected pollination and fertilization during flowering, and subsequent fruit setting and yield, the current result was confirmed by ‘Dashehari’ that was effected by weather and had unstable yield^42^. Similar to the response of pollen germination rate to temperature stress, the modified bilinear model described the response of pollen tube length under temperature stress. At the optimum temperature, the maximum length of pollen tube was from 51.2 μm of ‘Dashehari’ to 56.6 μm of ‘Jinhuang’, the average value of 53.7 μm. The difference and range of pollen tube length observed in current study were similar to ornamental pepper^43^ and soybean^44^ and other crops cultured in artificial pollen germination solution. In the results of Brassica napus, Young et al.^45^ pointed out that at high temperature, the poor fertility of pollen would lead to the decrease of pollen germination rate, which was the result of the loss of pollen moisture^46^; Pressman et al.^47^ suggested that high temperature affected the starch accumulation during pollen grains developed, which led to the decrease of the concentration of soluble sugar in mature pollen, so as to further reduce the pollen germination rate.

As for the adverse effects of temperature change on pollen germination, some researchers linked the response degree of different genotypes and even different species^48,49^, and tested the performance of pollen under different temperature stress^20,48^. Mango, widely cultivated in Southern China, often suffered from low temperature and rainy weather during flowering period in production, which led to abnormal meiosis of pollen mother cells, resulting in pollen fertilization and fruit drop^50^. Therefore, according to pollen germination rate and tube length CSRI value of different varieties of mango under different temperature stress, it was found that low temperature stress had a significant inhibitory effect on the growth of pollen tube, which was consistent to previous study^51^. On the contrary, high temperature stress had a greater destructive effect on mango pollen germination than that of low temperature by analyzing the changes of pollen germination rate of four mango varieties under different temperature stress^52^.

In order to compare the oxidative stress levels of different temperatures stresses on different genotypes of mango, the changes of enzymatic antioxidant activities and MDA content were measured. It is well known that enzymatic antioxidants such as SOD and POD can detoxify the excessive accumulation of ROS in pollen tubes caused by toxic stress^53–55^. In addition, when the content of ROS exceeds the threshold, lipid peroxidation occurs in both cell and organelle membrane, which had an adverse effect on normal cell function. MDA level was the final product of lipid peroxidation, which was often used as an indicator of ROS mediated cell membrane damage under temperature stress^32^. Gao et al.^56^ discussed that under temperature stress induction, a large increase in MDA content was also detected in the pollen tube of Pyrus pyrifolia. The effect of high temperature on enzyme activity and MDA content was greater than that of low temperature on mango genotype. ‘Apple mango’ had the largest change rate of SOD, POD activity and MDA content under low temperature and high temperature conditions. At the same time, considering CSRI value and other factors, it can be seen that ‘Apple mango’ was the most sensitive to low temperature and high temperature stress. In addition, ‘Renong No. 1’ had the smallest change rate of SOD, POD activity and MDA content under low temperature and high temperature conditions. Therefore, ‘Renong No. 1’ was the genotype with the strongest resistance to low temperature and high temperature stress.

At present, with the occurrence of more and more extreme climates around the world, the damage of extreme weather to the plant production process was more serious compared to the rise of the average temperature in the whole mango planting season^57^. Therefore, it was vitally important to identify and cultivate mango varieties those can tolerant extreme weather change^58^.

Current research collected mango pollen during mango blooming period, four varieties were treated with different temperature stress, and estimated the maximum values of pollen germination rate (PG), pollen tube length (PTL) and their basic temperature (T_min_, T_opt_ and T_max_) according to quadratic equation and modified bilinear model. These effects of temperature on the germination characteristics of mango pollen were explored by using cumulative stress response index (CSRI), POD, SOD and MDA, as well as determination on the differences of mango varieties.

In all four mango genotypes, pollen tubes were more tolerant to low temperatures stress than that to high temperatures stress. Among the four genotypes, the ‘Apple mango’ genotype was the most sensitive to low temperature and high temperature stress, and the ‘Renong No. 1’ genotype was the most resistant to low temperature and high temperature stress. In conclusion, different temperatures had great effects on the flowering, pollination, fertilization and fruit setting of mango of the same genotype, and there were also significant differences between different genotypes. Current research results were of great significance for the introduction of new mango varieties in different ecological regions, the cultivation and management of mango at the flowering stage and the breeding of new mango varieties.

## Materials and methods

### Pollen collection

Mango pollen was collected from mango field genebank, South Subtropical Crops Research Institute, Chinese Academy of Tropical Agricultural Sciences in March 2021 (Zhanjiang, China). In the blooming period (15–75% of the flowering number in total flowers), mango trees of each varieties were randomly selected. The fresh anthers that bloomed but pollen not scattered on the same day were put into the culture solution, shaken sufficiently and quickly brought back to the laboratory for observation and analysis under different temperature stress.

### Pollen germination and pollen tube length in vitro

1. Preparation of mango pollen culture solution: Mixed sucrose 150 g, boric acid 150 mg, calcium sulfate 6.0 mg, magnesium sulfate 100 mg, potassium nitrate 100 mg and polyethylene glycol 250 g, and dissolve to 1 L, which was heated and boiled to dissolve, then stored at normal temperature after cooled until used.
2. Pollen collection and pretreatment: the prepared culture solution was packed into 500 ul centrifuge tubes, from which picked 12–15 anthers randomly and fully vibrated to adhere the pollen to the culture solution.
3. Pollen culture in vitro at different temperatures: the centrifuge tubes were stored in incubators at 14 ℃, 16 ℃, 18 ℃, 20 ℃, 22 ℃, 24 ℃, 26 ℃, 28 ℃, 30 ℃, 32 ℃, 34 ℃ and 36 ℃ for 1 h, 3 h and 5 h, respectively, with continuous high and low temperature stimulation.
4. Determination on pollen germination rate under different temperature conditions: take a clean concave glass substrate, and then drop 1–2 drops of mixed solution into the concave for observation, and three replicates for each treatment. Randomly select five views for each repetition for observation on 200–500 pollen grains. Taken the pollen tube length exceeding the pollen grain diameter as the pollen germination standard, observe the number of germinated pollen and the total number of pollen under different temperature gradients with continuous stimulation for 1 h, 3 h and 5 h respectively, and count the germination rate and other pollen germination conditions. The calculation was carried out as the formula.
  $${\text{Q}} = \frac{w}{{\text{W}}} \times {\text{100\% }}$$
  In the formula: ${\text{Q}}$ is the germination rate; $w$ is the number of germinated pollen; ${\text{W}}$ is the total number of pollen;
5. For the length of pollen tube, three glass substrates were prepared, on which the length of about 50 germinated powder grains was measured by using optical microscope (Olympus bx-51).

### Curve fitting equation

In order to analyze the characteristics of pollen germination after temperature stress, linear and nonlinear regression models of temperature, maximum pollen germination rate and pollen tube length were established. In terms of R^2^ and contribution rate of root mean square deviation (RMSD) of observation and fitted values, the fitting results of linear and nonlinear regression models were compared. The pollen germination rate and pollen tube length were obtained according to the quadratic linear model and the modified bilinear model, as well as the basic temperature of all genotypes based on the fitting equation.

The parameters of quadratic equation and modified bilinear equation are estimated according to nonlinear regression program PROC NLIN^59^. In terms of quadratic model, the minimum temperature ($T_{\min }$), the optimum temperature ($T_{opt}$)) and the maximum temperature ($T_{\max }$) were estimated by equation. The formula for calculating the pollen germination rate and basic temperature was as follows Eqs. (1)—(4):

1 $$PG = a + bT - cT^{2}$$

2 $$T_{opt} = \frac{ - b}{{2c}}$$

3 $$T_{\min } = \frac{{ - b + \sqrt {b^{2} - 4ac} }}{2c}$$

4 $$T_{\max } = \frac{{ - b - \sqrt {b^{2} - 4ac} }}{2c}$$

In this formula, $PG$ was the pollen germination rate, $T$ was the actual temperature, a,b,c are the genotype specific constants generated by PROC NLIN in SAS.

In terms of the modified bilinear equation of pollen tube growth and basic temperature, the following formula was shown in Eqs. (5)–(7):

5 $$PTL = a + b_{1} (T - T_{opt}^{\prime } ) + b_{2} \times ABS(T_{opt} - T)$$

6 $$T_{\min }^{\prime } = \frac{{a + (b_{2} - b_{1} ) \times T_{opt} }}{{b_{1} - b_{2} }}$$

7 $$T_{\max }^{\prime } = \frac{{a - (b_{2} + b_{1} ) \times T_{opt} }}{{b_{1} + b_{2} }}$$

Of them,$T_{opt}^{\prime }$ was fitted by SAS, $T_{\min }^{\prime }$, $T_{\max }^{\prime }$ were determined by Eqs. (6) and (7).

### Cumulative stress response index (CSRI)

Taken pollen tube germination rate (GR) and pollen tube length (TL) of control group(c) and treatment croup (T) as indexes, the response of pollen tube to temperature stress was determined. The CSRI value was calculated using the following formula.

$$CSRI = \left( {\frac{{GR_{t} - GR_{c} }}{{GR_{c} }} + \frac{{TL_{t} - TL_{c} }}{{TL_{c} }}} \right) \times 100$$

### Determination of SOD and POD enzyme activity

SOD and POD enzyme reagent kit purchased from Suzhou Grace Biotechnology Co., Ltd. (Suzhou, China), measurement of SOD and POD enzyme activity according to the manufacturer's manual. SOD activity is often determined by riboflavin NBT method. The enzyme activity was determined according to the inhibition of superoxide dismutase on the reduction of nitroblue tetrazole (NBT) under light. The amount of enzyme required to inhibit the photochemical reduction of NBT by 50% is one enzyme activity unit (U)^53,60^. POD activity is often determined by guaiacol method^61^. Peroxidase activity was determined by measuring the absorbance change at 470 nm wavelength. In the calculation of enzyme activity, the increase of absorbance A470 value per minute by 0.1 is one enzyme activity unit (U).

### Determination of MDA content

MDA content was detected according to the method^62^. Weighed 0.5 g mango pollen, added 2 ml 5% TCA and a small amount of quartz sand, grind to the homogenate, added 8 ml TCA for further grinding, centrifuge the homogenate at 4000 r/min for 10 min, and the supernatant was the sample extract. Sucked 2 ml of supernatant, added 2 ml of 0.67% TBA solution and shake well. Put the test tube into boiling water bath and boiled for 10 min, took out the test tube and cooled it, and centrifuged for 15 min at 3000 r/min. The absorbance values at 532, 600 and 450 nm were measured with 0.67% TBA solution as blank.

### Data analysis

Excel 2019 and SPSS 26.0 version software was used for statistical analysis. The difference was compared by using ANOVA with a threshold P value of 0.05.

### Statement of research involving plants

The collection of plant material and all methods were carried out in accordance with relevant guidelines of National Field Genebank for Tropical Fruit (Zhanjiang, China).

## Data Availability

The data that support the findings of this study are available from the corresponding author on reasonable request.
